# Magnetodielectric detection of magnetic quadrupole order in Ba(TiO)Cu_4_(PO_4_)_4_ with Cu_4_O_12_ square cupolas

**DOI:** 10.1038/ncomms13039

**Published:** 2016-10-04

**Authors:** K. Kimura, P. Babkevich, M. Sera, M. Toyoda, K. Yamauchi, G. S. Tucker, J. Martius, T. Fennell, P. Manuel, D. D. Khalyavin, R. D. Johnson, T. Nakano, Y. Nozue, H. M. Rønnow, T. Kimura

**Affiliations:** 1 Division of Materials Physics, Graduate School of Engineering Science, Osaka University, Toyonaka 560-8531, Japan; 2 Laboratory for Quantum Magnetism, Institute of Physics, École Polytechnique Fédérale de Lausanne (EPFL), CH-1015 Lausanne, Switzerland; 3 Department of Physics, Tokyo Institute of Technology, Meguro-ku, Tokyo 152-8550, Japan; 4 ISIR-SANKEN, Osaka University, Ibaraki 567-0047, Japan; 5 Laboratory for Neutron Scattering and Imaging, Paul Scherrer Institut, CH-5232 Villigen, Switzerland; 6 ISIS facility, STFC Rutherford Appleton Laboratory, Chilton, Didcot OX11 0QX, UK; 7 Department of Physics, Graduate School of Science, Osaka University, Toyonaka 560-0043, Japan

## Abstract

In vortex-like spin arrangements, multiple spins can combine into emergent multipole moments. Such multipole moments have broken space-inversion and time-reversal symmetries, and can therefore exhibit linear magnetoelectric (ME) activity. Three types of such multipole moments are known: toroidal; monopole; and quadrupole moments. So far, however, the ME activity of these multipole moments has only been established experimentally for the toroidal moment. Here we propose a magnetic square cupola cluster, in which four corner-sharing square-coordinated metal-ligand fragments form a noncoplanar buckled structure, as a promising structural unit that carries an ME-active multipole moment. We substantiate this idea by observing clear magnetodielectric signals associated with an antiferroic ME-active magnetic quadrupole order in the real material Ba(TiO)Cu_4_(PO_4_)_4_. The present result serves as a useful guide for exploring and designing new ME-active materials based on vortex-like spin arrangements.

## Abstract (web summary)

Experimental knowledge of magnetoelectric (ME) activity from magnetic multipole moments has so far been limited. Here, Kimura *et al*. show that a magnetic square cupola cluster such as Cu_4_O_12_ realized in Ba(TiO)Cu_4_(PO_4_)_4_ is a promising structural unit carrying ME-active magnetic quadrupole moments.

## Graphical abstract

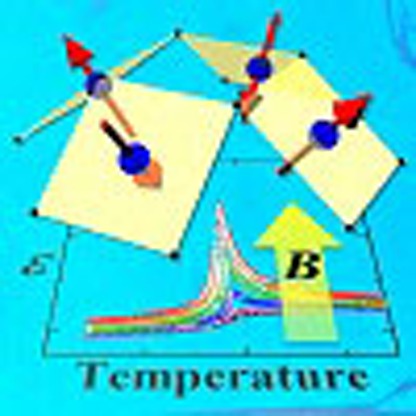

In magnetic materials, noncollinear spin arrangements often emerge when spins are placed in a particular lattice geometry such as geometrically frustrated lattices^1^. Symmetry-breaking properties of an additional multispin degree of freedom inherent in the noncollinearity can generate various anomalous magnetic phenomena. A well-known example is spiral-spin-driven ferroelectricity arising from vector spin chirality with broken space-inversion symmetry^2^,^3^,^4^,^5^. Another example is the magnetoelectric (ME) effect—magnetic field (*B*) control of electric polarization (*P*) and electric field (*E*) control of magnetization (*M*)—which originates from magnetic multipole moments that break both space-inversion and time-reversal symmetries^6^,^7^,^8^,^9^,^10^,^11^,^12^. Three types of symmetrically distinct ME-active multipole moments are known: the toroidal moment (**t**∝Σ_*n*_**r**_*n*_ × **S**_*n*_); the monopole moment (*a*∝Σ_*n*_**r**_*n*_·**S**_*n*_); and the magnetic quadrupole moment (*q*_*ij*_∝Σ_*n*_[*r*_*ni*_*S*_*nj*_+*r*_*nj*_*S*_*ni*_−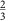
*δ*_*ij*_**r**_*n*_·**S**_*n*_]), where *n* represents a label of the spin **S**_*n*_ at the position vector **r**_*n*_ and *i*, *j* denote the *x*, *y* or *z* axis^7^,^10^,^13^,^14^. These quantities can be finite in a specific vortex-like spin arrangement. For example, the spin arrangements illustrated in Fig. 1a,b have the monopole moment *a* and the quadrupole moment 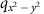
(=*q*_*xx*_−*q*_*yy*_), respectively, which therefore allow for the linear ME effect determined by the corresponding ME tensor (for more details, see Supplementary Note 1). Previous theoretical studies^4^,^15^ predict that a *B*-induced vortex deformation can generate finite *P* in toroidal and monopolar vortices (Fig. 1a), and also in the quadrupolar vortex as shown in (Fig. 1b), which is independent of the ME coupling mechanism. Of the three types of multipole moments, the uniform ordering of toroidal moments (ferrotoroidicity) and associated ME activities have been experimentally probed via various techniques^9^,^12^,^16^,^17^,^18^,^19^. In particular, an observation of ferrotoroidic domain structure^9^ and its hysteretic switching by crossed magnetic and electric fields^12^,^18^ have led researchers propose ferrotoroidicity to be a fourth form of primary ferroic orders in the fundamental scheme based on the order-parameter symmetries with respect to the space-inversion and time-reversal operations. The ME activity of the other magnetic multipole moments, however, has never been established experimentally. Here we propose a strategy for the realization of these ME-active multipole moments in a material starting from a simple magnetic cluster.

The magnetic cluster considered here is illustrated in Fig. 1c,d. It consists of four transition metal ions forming a spin plaquette and twelve coplanar ligands such as oxygen. This is a fragment of a very common lattice seen in many inorganic materials including the high-*T*_c_ cuprate superconductors^20^ and infinite-layer iron oxides^21^. If we introduce a single-ion anisotropy (or exchange anisotropy) normal to the metal-ligand plane and simple ferromagnetic (FM) or antiferromagnetic (AFM) interactions between the spins, the FM and AFM spin-plaquette show an all-up (or all-down) and an up-down-up-down structure, respectively. Both are ME inactive, because they have neither toroidal, monopole nor quadrupole moments. To induce ME activity, we introduce a buckling deformation that transforms the cluster geometry to one of the Johnson solids^22^ known as square cupola (Fig. 1e,f), while assuming that the anisotropy and exchange interactions remain unchanged. With this buckling, the FM spin plaquette has monopole components in addition to ferromagnetic components, while the AFM spin plaquette has magnetic quadrupole components. Therefore, in principle, both square cupola clusters should exhibit ME activity.

To experimentally verify this ME design strategy, we have searched for a real material that comprises magnetic square cupola clusters. The candidate compound found is Ba(TiO)Cu_4_(PO_4_)_4_, a recently synthesized magnetic insulator crystallizing in a chiral tetragonal structure with space group *P*42_1_2 (ref. 23). The crystal structure is illustrated in Fig. 2a,b. The magnetic properties are dominated by Cu^2+^ ions (*S*=1/2) with square planar coordination of oxygen ions. Notably, the crystal structure comprises an irregular Cu_4_O_12_ square cupola cluster formed by four corner-sharing CuO_4_ planes—that is, an experimental realization of Fig. 1e,f. Two types of square cupola clusters, A (upward) and B (downward), distinguished by their direction with respect to the *c* axis, align alternatingly in the *ab*-plane to form a layered structure, which we call a square cupola layer. Importantly, the nature of the square cupola is expected to be preserved in this compound because neighbouring square cupola clusters do not share any oxygen, suggesting weak inter-cluster couplings (Fig. 2b). These features make this material suitable for testing our ME design strategy. Therefore, we have studied magnetic and ME properties of Ba(TiO)Cu_4_(PO_4_)_4_, demonstrating that the magnetic structure is describable by an antiferroic magnetic quadrupole order and, moreover, that an associated ME activity is manifested in the magnetodielectric properties. These results successfully verify our ME design strategy.

## Results

### Magnetic properties

The temperature (*T*) dependence of magnetization (*M*) divided by *B* (*χ*≡*M*/*B*) applied along the [100] and [001] axes is shown in Fig. 2c. Fits to the high-temperature data (*T*>100 K) of *χ*_[100]_ and *χ*_[001]_ using the Curie–Weiss law yield effective moments of 1.92(1) *μ*_B_/Cu and 1.96(1) *μ*_B_/Cu, respectively, which are typical for Cu^2+^ ions, and AFM Weiss temperatures (*θ*_CW_) of −33.2(6) and −30.1(2) K. On cooling, a broad maximum appears at around 17 K, followed by a clear anomaly at *T*_N_=9.5 K, below which *χ* shows an anisotropy. Because of the two-dimensional (2D) nature of the square cupola layers due to the separation by nonmagnetic Ba and Ti layers, the broad maximum suggests a development of short-range correlation within each cluster and/or 2D layer. The anomaly at *T*_N_=9.5 K indicates the onset of AFM long-range order due to weak inter-layer couplings. Notably, both *χ*_[100]_ and *χ*_[001]_ remain finite at the lowest temperature measured, which indicates that the magnetic structure is not a simple collinear antiferromagnet. No metamagnetic transition is observed up to *B*=7 T, as demonstrated by approximately linear magnetization curves at *T*=1.8 K (Fig. 2d). The AFM transition is also evidenced by a peak in specific heat *C_P_*, as shown in Fig. 2e.

**Figure 1:**
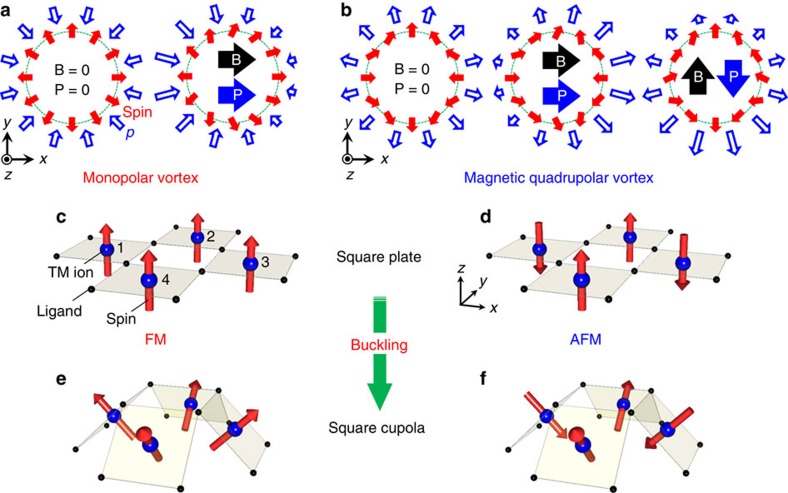
Conceptual design route of ME-active spin cluster. (**a**,**b**) Schematic illustrations of monopolar (**a**) and magnetic quadrupolar (**b**) vortices in the absence and presence of a magnetic field *B* (solid black arrow). The red solid, blue open and blue solid arrows represent the orientation of spin, the local electric polarization *p* and macroscopic electric polarization *P*, respectively. (**c**,**d**) A square planar cluster composed of ferromagnetically (FM) (**c**) and antiferromagnetically (AFM) (**d**) coupled four spin plaquette, respectively. Blue and black balls represent the transition metal (TM) ions (*n*=1–4) and ligands, respectively. (**e**,**f**) Square cupola clusters after buckling the cluster in **c** and **d**, respectively. The FM square cupola cluster in **e** has the monopole moment 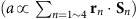 while the AFM square cupola cluster in **f** has the magnetic quadrupole moment 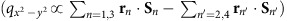, where **r**_*n*_ is a position vector of the TM ion *n* from the centre of the four TM ions and **S**_*n*_ is a spin moment.

For microscopic characterization of the magnetic properties, we use neutron diffraction. As depicted in Fig. 3a, below 9.5 K we observe new Bragg peaks emerging corresponding to magnetic ordering of moments. The magnetic reflections can be indexed using a single propagation wave vector **k**=(0, 0, 0.5), which corresponds to a doubling of the unit cell along the [001] direction. Symmetry analysis indicates that the magnetic representation can be decomposed into Γ_mag_=3Γ_1_+3Γ_2_+3Γ_3_+3Γ_4_+6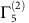, where the irreducible representation 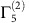 is 2D and the others are one-dimensional. The best-fit magnetic structure obtained by the Rietveld refinement with the magnetic *R*-factor of 11.5% (Γ_3_ irreducible representation) at 1.5 K is illustrated in Fig. 3b for a single square cupola layer. The magnetic structure is noncollinear, with the moments tilted away from the *c* axis in such a way that they are approximately perpendicular to the CuO_4_ plane. Notably, in each Cu_4_O_12_ square cupola (Fig. 3c), the *c* axis components of magnetic moments align in the up-down-up-down manner while the *ab*-plane components rotate by 90°. This demonstrates an experimental realization of the AFM square cupola considered in Fig. 1b, which carries a magnetic quadrupole moment composed of an almost pure 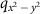 component. Moreover, the 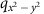 components in every Cu_4_O_12_ square cupola align uniformly, suggesting that the magnetic structure can be considered as a uniform order of mangnetic quadrupole moments within the *ab*-plane. Further details on this quadrupole order can be found in Supplementary Fig. 1 and Supplementary Note 2. An ordered moment of 0.80(3)*μ*_B_ is found on each Cu site. The details of refinement are provided in Supplementary Figs 2–4, Supplementary Tables 1 and 2, and Supplementary Note 3.

### Dominant exchange interactions

The quadrupole-based description of the magnetic structure is valid if intraplaquette exchange interactions dominate over interplaquette ones. To examine this point, we have performed inelastic neutron-scattering measurements. Colour plot of inelastic neutron spectra at *T*=2 K is shown in Fig. 3d. We observe two strong, flat bands of intensity close to 3.2 and 4.2 meV (Fig. 3e). This is consistent with the energy scale estimated from *θ*_CW_≈−30 K. In addition, we find branches dispersing from magnetic zone centres with a gap energy of around 1 meV. On warming to 30 K, shown in Fig. 3e, we find that both the gapped branches and the flat bands of intensity disappear as would be expected for excitations, which are magnetic in origin. Further measurements using *E*_i_=12.1 meV did not reveal any additional excitations. The observed inelastic spectrum is consistent with strongly coupled plaquettes with weak inter-plaquette interactions and an anisotropy, such as from Dzyaloshinskii–Moriya interaction, which is symmetrically allowed in this material. The former would result in dispersive collective excitations and the latter create a spin-gap. Therefore, Ba(TiO)Cu_4_(PO_4_)_4_ could potentially be an exciting system to examine the cross-over from local quantum levels to dispersive spin-waves of coupled spin clusters such as in Cu_2_Te_2_O_5_(Cl,Br)_2_ (ref. 24).

**Figure 2:**
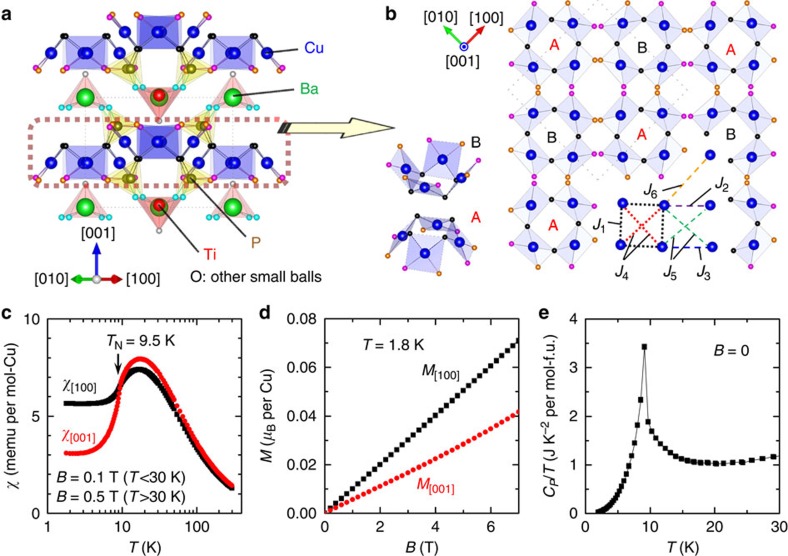
Crystal structure and magnetic properties of Ba(TiO)Cu_4_(PO_4_)_4_. (**a**) Crystal structure of Ba(TiO)Cu_4_(PO_4_)_4_ viewed along the [110] direction. The grey dotted line represents a unit cell. (**b**) Square cupola layer viewed along the [001] direction. Two types of Cu_4_O_12_ square cupola clusters, A (upward) and B (downward), are shown. Intraplaquette (*J*_1_ and *J*_4_) and interplaquette (*J*_2_, *J*_3_, *J*_5_, and *J*_6_) exchange interactions are denoted by dotted and dashed lines, respectively. (**c**) Temperature dependence of magnetic susceptibility for the field along the [100] (black square) and [001] (red circle) directions. (**d**) Magnetization curves at 1.8 K. (**e**) Temperature dependence of specific heat divided by temperature.

Further insights to exchange interactions are provided by density functional theory (DFT) calculations. The electronic structure of Ba(TiO)Cu_4_(PO_4_)_4_ was calculated by using GGA+*U* method^25^,^26^, where the on-site Coulomb repulsion *U*_eff_ was set to 4 eV. We have calculated the magnetic exchange coupling constants *J*_*k*_ (*k*=1–6) between the spins (Fig. 2b). Here positive (negative) *J* represents FM (AFM) interactions. We find that the strongest interaction is the nearest-neighbour intraplaquette *J*_1_=−3.0 meV, followed in order by orthogonal interplaquette interaction *J*_5_=−0.7 meV and orthogonal intraplaquette *J*_4_=−0.5 meV. The other interactions are one order of magnitude smaller than *J*_1_ (*J*_2_=−0.2 meV, *J*_3_=0.2 meV and *J*_6_=−0.1 meV). From these coupling constants the Weiss temperature is calculated to be −31 K, which is comparable to the experimental value of *θ*_CW_≈−30 K. We have also calculated *J*_*k*_ with different values of *U*_eff_ (0 and 7 eV) and confirmed that the relative strengths of *J*_*k*_ is almost insensitive to *U*_eff_. Thus, our experimental and theoretical studies establish that the present material is a weakly coupled plaquette antiferromagnet, which means that the quadrupole-based description of the magnetic structure is valid. Furthermore, a preliminary spin-wave calculation^27^ using the exchange parameters (*J*_1_–*J*_6_) obtained from DFT calculations gives a qualitative agreement to the measured inelastic neutron spectrum, which confirms the consistency between the experiments and the DFT results (Supplementary Fig. 5). However, because of a relatively large number of exchange paths which must be taken into account, single-crystal study is necessary for a detailed comparison of inelastic neutron spectrum and a model based on DFT parameters.

### Magnetodielectric effect

The task is now to experimentally confirm the ME activity of these quadrupole moments. Usually, it can be easily probed through the measurements of *B*-induced *P* or *E*-induced *M*. In the present case, however, it is not straightforward because, as indicated by the 00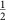 magnetic wave vector, the quadrupole moments are antiferroically coupled along the *c* axis, which results in the cancellation of the associated linear ME response. Indeed, our high-precision pyroelectric current measurement (<0.1 pA) at a high-*B* (<9 T) does not show any signal indicative of a macroscopic *B*-induced *P* near *T*_N_. Nonetheless, we observe a clear signature for the ME-activity in the dielectric constant (*ɛ*), as shown in the following.

Figure 4a shows the *T*-dependence of *ɛ* along the [100] direction (*ɛ*_[100]_) measured at selected *B* applied along the [100] direction (*B*_[100]_). While *ɛ*_[100]_ shows only a slight anomaly at *T*_N_ in the absence of *B*_[100]_, the application of *B*_[100]_ induces a divergent peak towards *T*_N_. Together with the absence of *P*, this result suggests the onset of the *B*-induced antiferroelectric (AFE) order, which is similar to the *B*-induced ferroelectric order observed in linear ME materials such as Cr_2_O_3_ (ref. 28). To quantitatively analyse the data, we define the *B*_[100]_-induced component in *ɛ*_[100]_ as Δ*ɛ*_[100]_(*B*_[100]_)≡*ɛ*_[100]_(*B*_[100]_)−*ɛ*_[100]_(0). The inset of Fig. 4a shows Δ*ɛ*_[100]_(*B*_[100]_) divided by the square of *B*_[100]_, Δ*ɛ*_[100]_/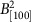, as a function of a reduced temperature, *t*≡(*T*−*T*_N_)/*T*_N_. Strikingly, all the data approximately collapse onto a single curve, meaning that Δ*ɛ*_[100]_(*B*_[100]_) is proportional to the square of *B*_[100]_. This scaling behaviour of the *B*-induced divergent peak is characteristic in AFM linear ME materials such as MnTiO_3_, which is accounted for on the basis of Landau free energy expansion involving the linear ME coupling term^29^. No divergent behaviour is observed for *ɛ*_[100]_ in *B*_[010]_ and *B*_[001]_, and for *ɛ*_[001]_ in all *B* directions. (Supplementary Fig. 7).

## Discussion

Because different ME-active multipole moments (**t**, *a* and *q*_*ij*_) lead to different forms of the ME tensor (Supplementary Fig. 6 and Supplementary Note 1), identifying the ME tensor in Ba(TiO)Cu_4_(PO_4_)_4_ is crucial to verify that the observed ME response originates from the ME activity of the quadrupole moments. To this end, we first discuss the observed ME response in terms of the linear ME effects in each square cupola layer. According to the transformation properties of the present Γ_3_ magnetic structure (Supplementary Table 2), the magnetic point group symmetry of each square cupola layer is 4′22′, which breaks both the space-inversion and time-reversal symmetries and therefore allows for a linear ME effect given by the ME tensor^30^


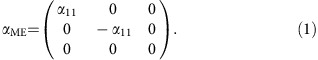


This ME tensor predicts that the application of *B*_[100]_ induces *P* along the [100] direction (*P*_[100]_). In addition, the 00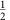 magnetic wave vector indicates that the sign of the ME tensor of the neighbouring layers is opposite to each other. This means that the system exhibits a *B*_[100]_-induced AFE behaviour along the [100] direction, consistent with the observed ME response. Note that this ME tensor allows, in principle, a macroscopic 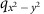-type quadrupole moment within the *ab*-plane (Supplementary Fig. 6), which is consistent with the uniform alignment of 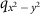 quadrupole moments on the individual square cupolas. Consistently, our group theory analysis in the framework of Landau theory of phase transitions^31^,^32^,^33^ shows that the quadrupole order can lead to the same ME effect as expected from the ME tensor in equation (1). Details of the analysis are provided in Supplementary Fig. 8, Supplementary Table 3 (refs 34, 35, 36, 37) and Supplementary Note 4.

**Figure 3:**
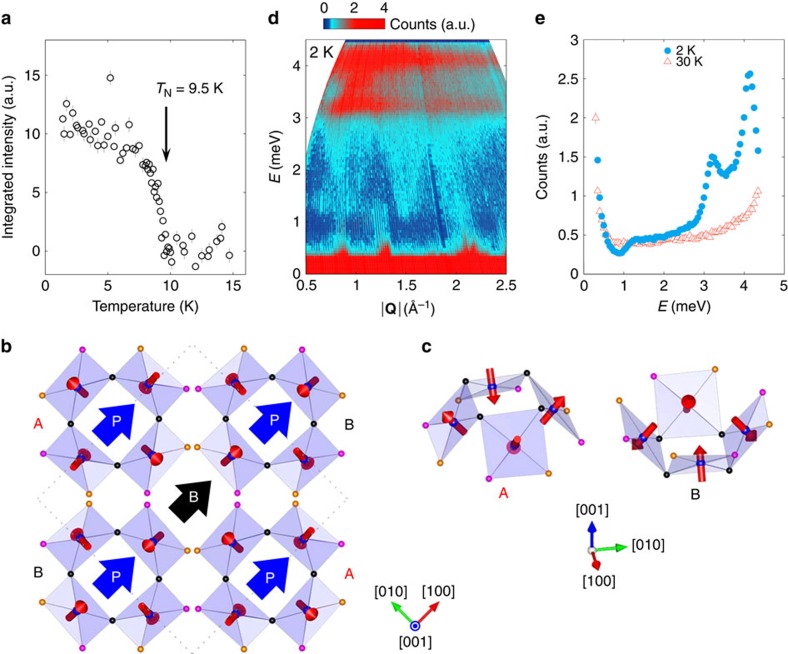
Magnetic structure and magnetic excitations probed through neutron-scattering experiments. (**a**) Temperature evolution of the sum of the integrated intensities of the 10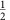, 11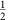 and 21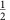 magnetic reflections (open circles). (**b**) Magnetic structure of a single square cupola layer. Expected direction of electric polarization *P* in each square cupola induced by a magnetic field (*B*) along the [100] direction is indicated. (**c**) Detailed configuration of magnetic moments in square cupolas A and B. (**d**) Inelastic neutron powder intensity map at 2 K as a function of momentum |**Q**| and energy *E* transfer. Measurements were recorded from a powder sample using incident neutron energy of 6 meV. (**e**) A constant-|**Q**| cut integrated between 1.3<|**Q**|<1.6 Å^−1^ at 2 and 30 K.

Next, we associate the observed result with the ME activity of individual magnetic quadrupole moments. As schematically illustrated in Fig. 1b, while no net *P* emerges from the magnetic quadrupole moment at *B*=0, the application of *B* along the *x* or *y* axis induces *P* parallel or antiparallel to the applied *B*, where the local principal axes parallel to the inward and outward spins are taken as the *x* and *y* axis, respectively. By applying this scenario to the single square cupola layer of the present system, we find that under *B*_[100]_ all the quadrupole moments generate *P* approximately parallel to the *B* direction, resulting in macroscopic *P*_[100]_ in the single square cupola layer (Fig. 3b). Although a small diagonal component of local *P* may be generated in each quadrupole moment due to the slight deviation of the inward spin direction from the [100] direction, this component cancels out in neighbouring quadrupole moments because of the symmetry. The direction of *B*-induced *P* is again consistent with that of the observed ME response and the ME tensor in equation (1). Note that there must be some contributions to the ME activity from the interplaquette interactions. However, the leading exchange interaction is the intraplaquette *J*_1_, which provides a reasonable basis for the interpretation of the observed ME activity in terms of the ME activity of individual square cupolas. Owing to an AFM stacking of quadrupole moments along the [100] direction, the induced *P* aligns antiferroelectrically along the [001] direction.

**Figure 4:**
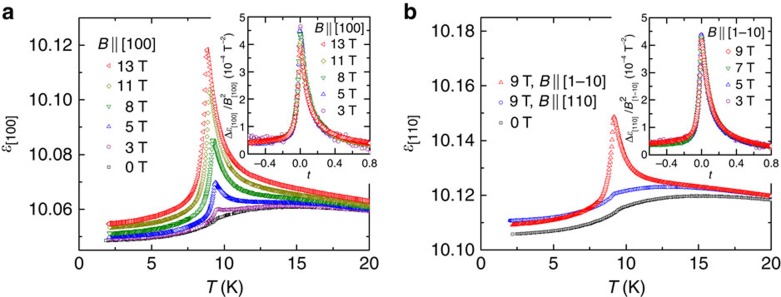
Magnetodielectric properties. (**a**) Temperature dependence of the dielectric constant along the [100] direction (*ɛ*_[100]_) in various magnetic fields applied along the [100] direction (*B*_[100]_). The inset shows the *B*_[100]_-induced change Δ*ɛ*_[100]_(*B*_[100]_)≡*ɛ*_[100]_(*B*_[100]_)−*ɛ*_[100]_(0 T) divided by the square of *B*_[100]_, Δ*ɛ*_[100]_/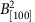, as a function of a reduced temperature, *t*≡(*T*−*T*_N_)/*T*_N_. (**b**) Temperature dependence of *ɛ*_[110]_ in *B*=0, *B*_[110]_=9 T and *B*_[1–10]_=9 T. The inset shows the Δ*ɛ*_[110]_/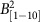 as a function of *t*.

Furthermore, on the basis of the ME tensor given by equation (1) as well as the ME activity of individual magnetic quadrupole moments (Fig. 1b), one can expect that *P*_[110]_ is induced by the application of *B*_[1–10]_ but not by *B*_[110]_, and the magnitude of *B*_[1–10]_-induced *P*_[110]_ is the same as that of *B*_[100]_-induced *P*_[100]_. This expectation is experimentally demonstrated in the effects of *B*_[1–10]_ and *B*_[110]_ on *ɛ*_[110]_ (Fig. 4b), together with the scaling behaviour of *ɛ*_[110]_ with the magnitude of Δ*ɛ*_[110]_/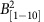 comparable to that of Δ*ɛ*_[100]_/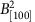 (Fig. 4b, inset). Thus, the observed AFE behaviour can be nicely explained in terms of the ME activity of quadrupole moments and their antiferroic alignment.

Finally, we discuss a possible microscopic origin for the observed ME activity. The previous theoretical work demonstrated that a magnetostriction mechanism via non-relativistic superexchange interactions can generate large ME effects in monopolar and toroidal vortices^15^. Applying the similar discussion to the present quadrupolar vortex on a square cupola, we have found that the magnetostriction mechanism can also lead to the *B*-induced *P*, and the direction of *B* and *P* is consistent with the experimental observation (Supplementary Fig. 9). However, because other known mechanisms associated with a relativistic spin orbit interaction, for example, the *d*–*p* hybridization mechanism^38^ and the spin-current^3^ (or inverse DM^5^) mechanism, can also be possible. Therefore, more detailed understanding of the microscopic mechanism for the ME activity is left for future study.

To conclude, we propose a magnetic square cupola cluster as a promising structural unit that carries the ME-active multipole moments, and confirm this idea by magnetodielectrically detecting the ME activity of the magnetic quadrupole moments in a real material. This result indicates that ME activity arising from magnetic multipoles can also be found in other square cupola based materials (for example, Na_5_*A*Cu_4_(AsO_4_)_4_Cl_2_ (*A*=Rb, Cs)^39^ and [NH_4_]Cu_4_Cl(PO_3_F)_4_ (ref. 40)). In particular, the monopolar vortex, which can appear in the FM square cupola (Fig. 1a), is interesting because it possesses a ferromagnetic component controllable by an electric field through the ME coupling. Another aspect that deserves attention in the present study is the discovery of the ME response due to the antiferroic ME-active multipole order. For example, this ME response allows for an *E*- or *B*-induced finite-*q* magnetization or electric polarization, which might be useful for future nanoscale spintronics. Moreover, the present discovery demonstrates that dielectric constant measurements can provide a rather straightforward, macroscopic signature for antiferroic order of any types of ME-active multipole moments. This is an important finding to explore a new state of matter such as a recently discussed antiferromonopolar state and antiferrotoroidic state^14^.

## Methods

### Sample preparation and characterization

Single crystals of Ba(TiO)Cu_4_(PO_4_)_4_ were grown by the flux method^23^. Powder X-ray diffraction measurements on crushed single crystals confirmed a single phase. The crystals were oriented using Laue X-ray diffraction. The crystal structures displayed in this article were drawn using VESTA software^41^. Magnetization (*M*) measurements down to temperature (*T*) of 1.8 K and magnetic field (*B*) up to 7 T were performed using a commercial superconducting quantum interference device magnetometer (Quantum Design MPMS3). The specific heat (*C*_*P*_) was measured down to 2 K by a thermal relaxation method using a commercial calorimeter (Quantum Design PPMS). For dielectric measurements, single crystals were cut into thin plates and subsequently silver electrodes were vacuum deposited on the pair of widest surfaces. The dielectric constant *ɛ* was measured using an *LCR* meter at an excitation frequency of 100 kHz. Pyroelectric current was measured by an electrometer (Keithley 6517) to monitor electric polarization.

### Neutron-scattering experiments

Neutron diffraction measurements were performed on a powder sample using the time-of-flight neutron diffractometer WISH at ISIS^42^ and the DMC diffractometer at the SINQ spallation source^43^. Magnetic and nuclear structure refinements were performed using FullProf^44^. The inelastic neutron-scattering measurements were carried out on a 17 g powder sample using the time-of-flight spectrometer FOCUS at the SINQ spallation source^45^. Using incident neutron energies of *E*_i_=6 and 12.1 meV, the energy resolution in the energy transfer range of interest was ∼0.2 and 0.7 meV, respectively.

### DFT calculations

DFT calculations were performed to estimate the magnitude of dominant magnetic interactions. The VASP (Vienna *ab initio* simulation package)^46^,^47^,^48^,^49^ was used with a projector-augmented wave basis set^50^,^51^. The electronic exchange and correlation were described by the Perdew–Burke–Ernzerhof generalized gradient approximation (PBE-GGA)^25^. The DFT+*U* method^26^ was used for correction for strongly correlated Cu-3*d* states. We first calculated the electronic structure of Ba(TiO)Cu_4_(PO_4_)_4_ with the experimental crystal structure. Then we obtained the total energy differences among several magnetic phases with different spin structures by performing spin-constrained DFT calculations. The magnetic exchange coupling constants *J*_*k*_ were estimated as the best fit for the energy differences within an effective classical Heisenberg model. Our model Hamiltonian is defined as follows:


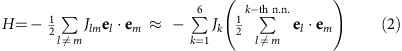


Here 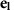 is a unit vector that point to the direction of the spin at site *l* and *J*_*lm*_ the effective coupling constant between sites *l* and *m*. The factor of 1/2 removes double counting. *J*_*k*_ is the average of *J*_*lm*_ taken over the *k*-th nearest-neighbour spin pairs. Note that the magnitude of spin (ideally *S*=1/2 for Cu^2+^ ions) is renormalized in *J*_*k*_ and that the summation over *k* terminates at 6 as we consider up to *J*_6_ (Fig. 2b).

### Data availability

The data that support the findings of this study are available from the corresponding author on request.

## Additional information

**How to cite this article:** Kimura, K. *et al*. Magnetodielectric detection of magnetic quadrupole order in Ba(TiO)Cu_4_(PO_4_)_4_ with Cu_4_O_12_ square cupolas. *Nat. Commun.* **7,** 13039 doi: 10.1038/ncomms13039 (2016).

## Supplementary Material

Supplementary Information Supplementary Figures 1-9, Supplementary Tables 1-3, Supplementary Notes 1-4 and Supplementary References
