# Supplementary material for: Magnetodielectric detection of magnetic quadrupole order in Ba(TiO)Cu4(PO4)4 with Cu4O12 square cupolas
Source: Nat Commun. 2016 Oct 4;7:13039. doi: 10.1038/ncomms13039 (PMC5059463; doi:10.1038/ncomms13039)
Supplement: Supplementary Information — Supplementary Figures 1-9, Supplementary Tables 1-3, Supplementary Notes 1-4 and Supplementary References [file ncomms13039-s1.pdf]

Supplementary Figures

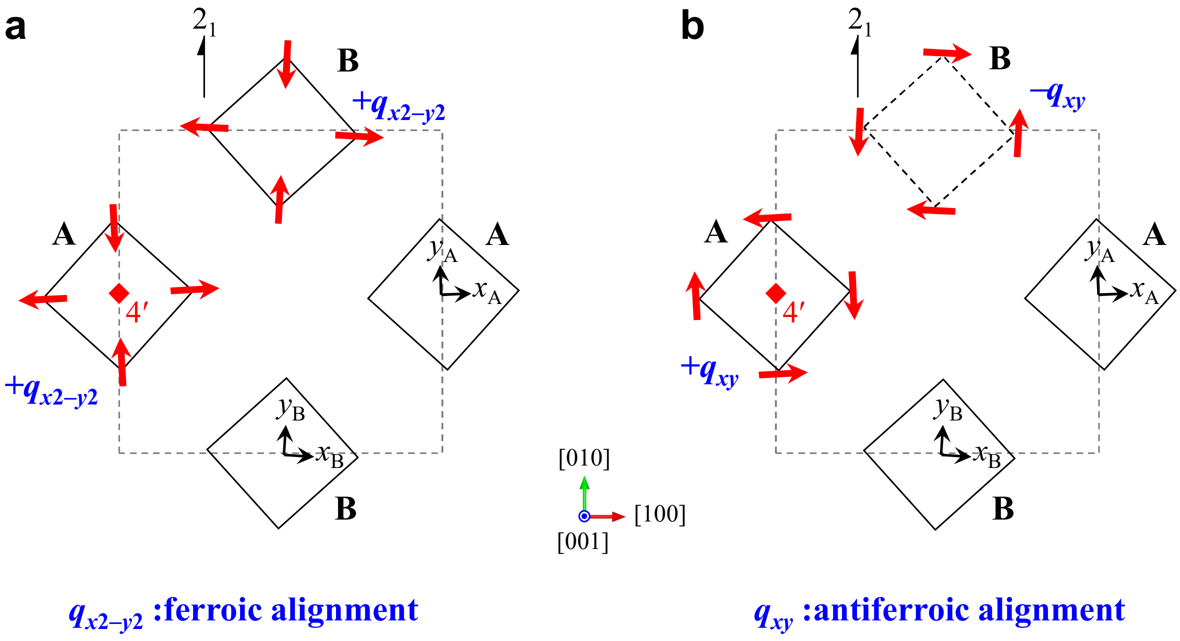

Supplementary Figure 1: Alignment of two types of quadrupole moments. a,**b**, The alignment of the q_{x2-y2} (**a**) and q_{xy} (**b**) quadrupole moments on a square cupola within the *ab*-plane. These quadrupoles are allowed by the 4′ symmetry located at the center of each square cupola. One of the 2_1 symmetry elements that connect square cupola A and B is also shown. The red arrows indicate the *ab*-plane components of the magnetic moments on Cu ions. The dashed outline shows the crystallographic unit cell. The local coordinates (*x, y*) for the square cupola A and B are also given.

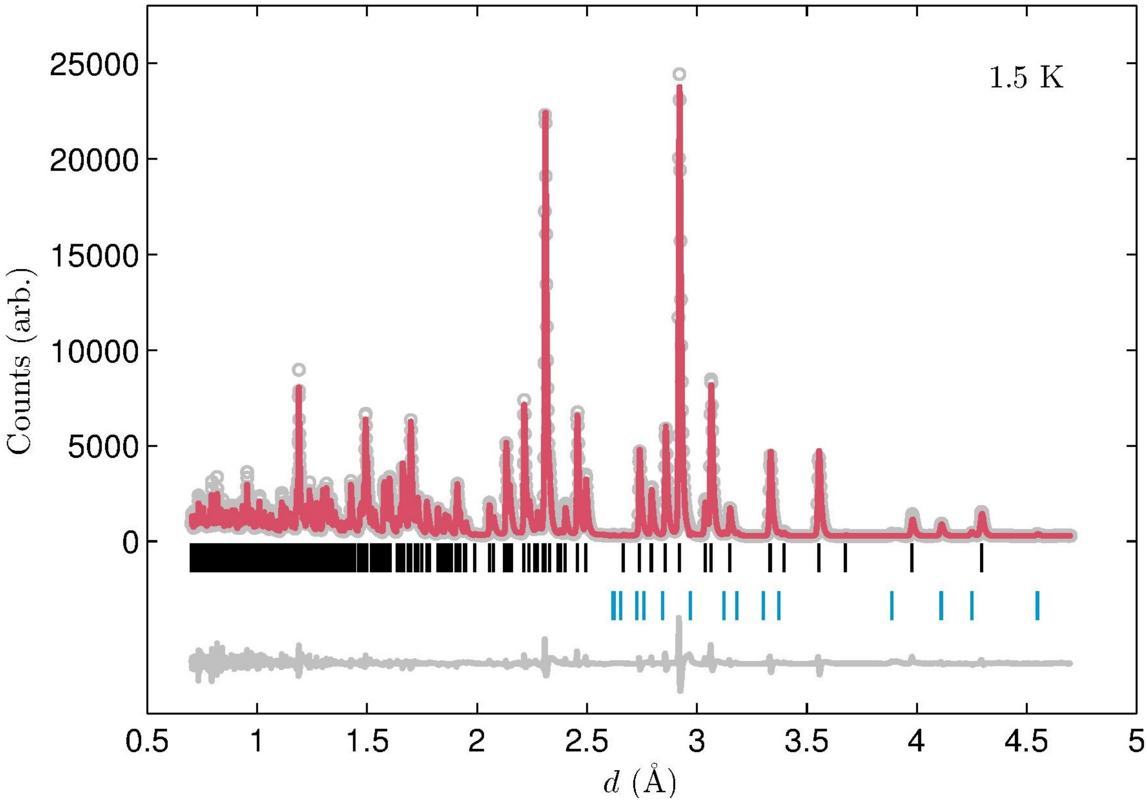

Supplementary Figure 2: Neutron diffraction pattern of $Ba(TiO)Cu_4(PO_4)_4$ at 1.5 K. Open circles denote the data and the red line represents the fit to the structural and magnetic models described in the text. The refinement for this pattern has reliability factors of R_p = 7.0% and R_{wp} = 7.2%. The position of nuclear and magnetic Bragg peaks is shown by black and blue vertical lines, respectively. The gray line plotted at the bottom shows the difference between the simulation and data.

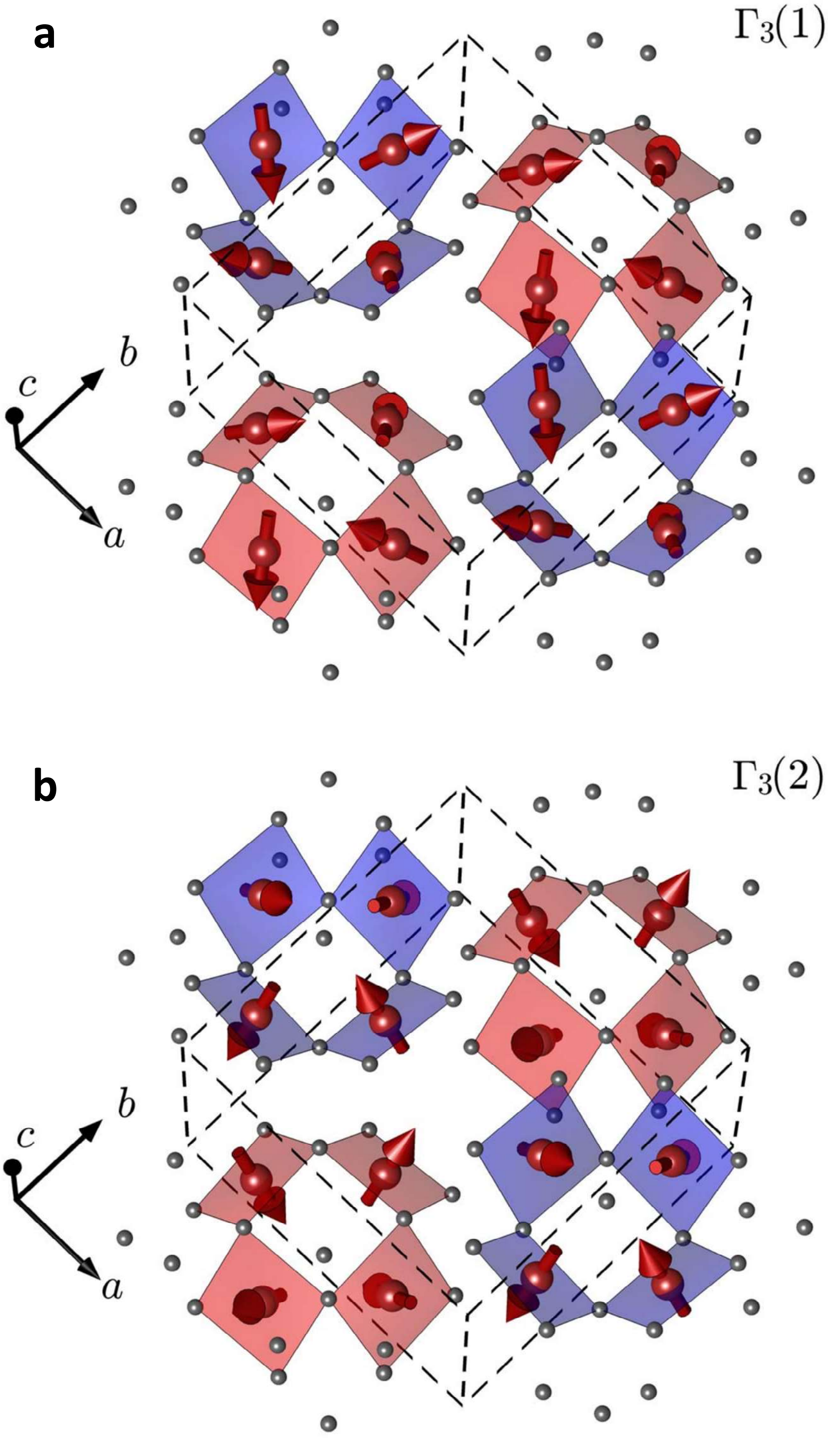

Supplementary Figure 3: Solutions for the magnetic structure in $Ba(TiO)Cu_4(PO_4)_4$ described in the text. The Cu and O ions are depicted by red and gray spheres, respectively. Other atoms are not shown. The CuO_4 planes within up/down square cupola clusters are shown in red and blue. The dashed outline shows the crystallographic unit cell.

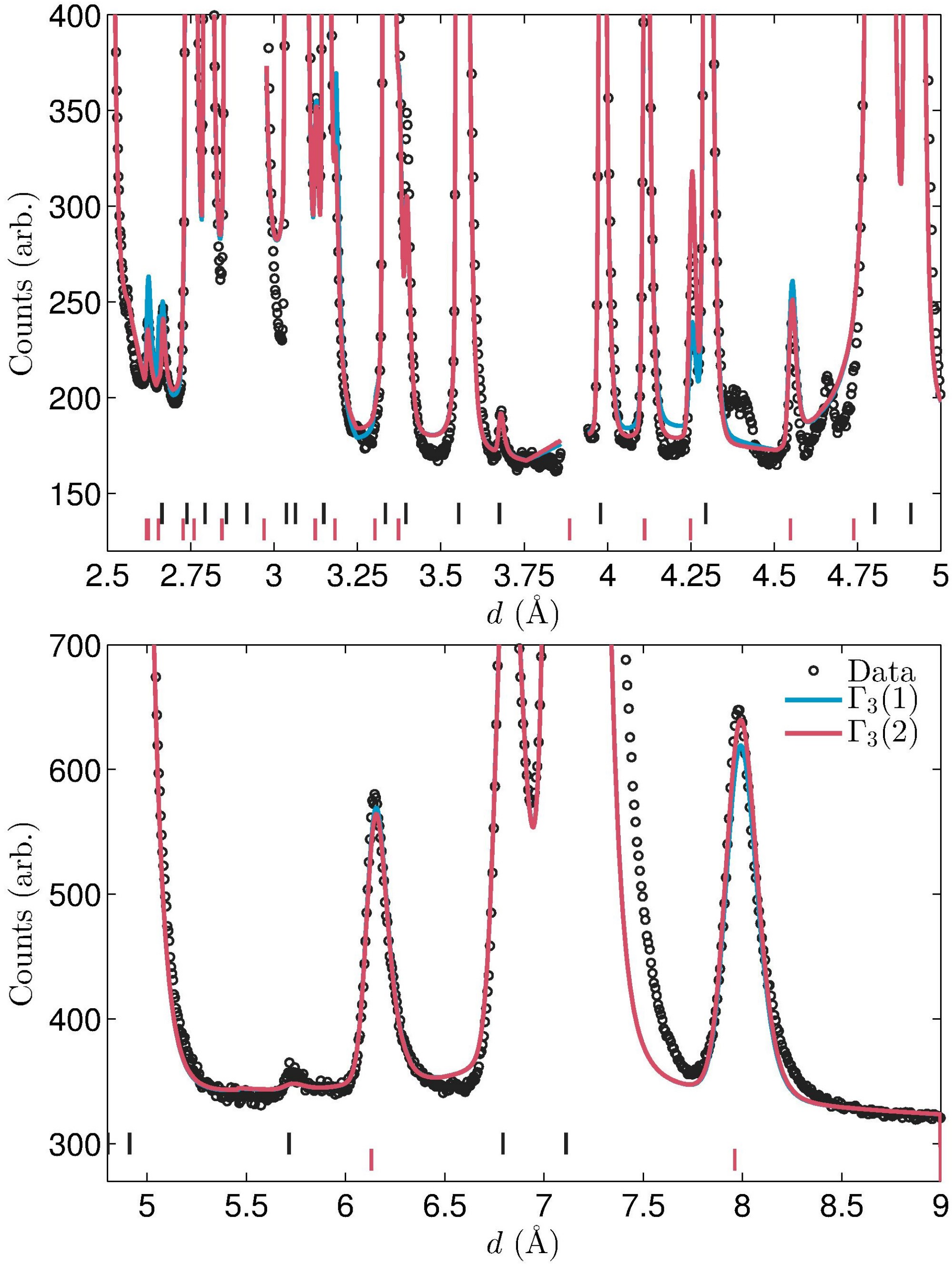

Supplementary Figure 4: Measured diffraction patterns on WISH of $Ba(TiO)Cu_4(PO_4)_4$ at 1.5 K. We present data from two different banks with different d-range and resolution. The simulations based on models $\Gamma_3(1)$ and $\Gamma_3(2)$ of the magnetic structure are presented. The position of nuclear Bragg peaks are denoted by black vertical lines. The magnetic reflections are indicated by the red vertical lines. The magnetic R-factors (R_{mag}) for $\Gamma_3(1)$ are (26.2%, 7.6%) and for $\Gamma_3(2)$ are (10.6%, 4.3%), where the first R_{mag} is given for the pattern $2.5 < d < 5$ Å and second for $5 < d < 9$ Å. Traces of impurities, can be resolved around 4.3 and 4.7 Å. Some magnetic reflections which were overlapping with nuclear or impurity scattering were removed from the fit such as around 3.8 Å.

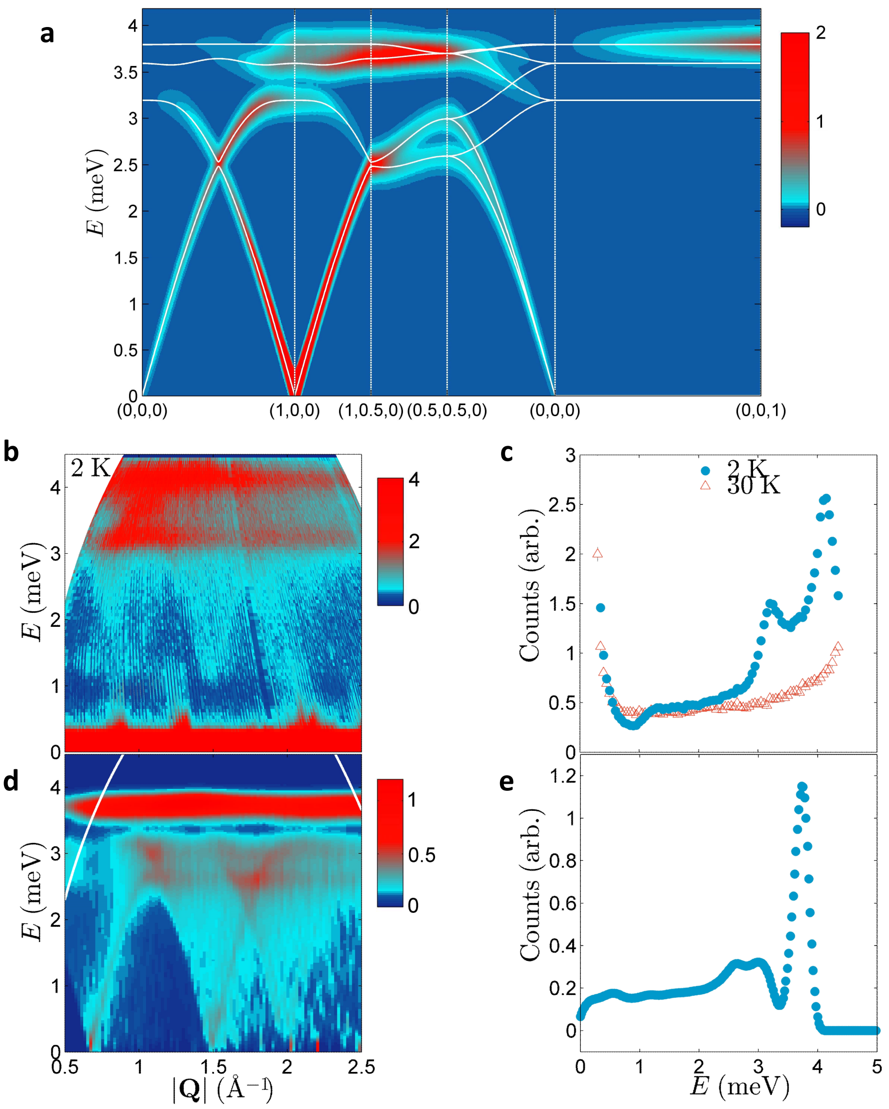

Supplementary Figure 5. Comparison of measured and calculated magnetic spectra. a, Calculated spin-wave dispersion along high-symmetry directions in reciprocal space using the exchange parameters ($J_1 \sim J_6$) obtained from DFT calculations. Calculations were performed using the SpinW package[1]. Solid lines represent the magnon modes and colors indicate the neutron scattering cross-section. An energy broadening of 0.2 meV was applied to the simulations. As the model does not contain any anisotropy terms, the dispersion is gapless. **b,c,** Measured powder-averaged magnetic spectrum (**b**) and integrated intensity between $1.3 < E < 1.6$ $Å^{-1}$ (**c**). **d,e,** Corresponding simulations. As expected from (**a**), a strong flat band of intensity around 3.8 meV in (**d**) originates from the optic modes at the top of the dispersion. Another band of scattering in (**d**) is found around 2.8 meV. This originates at the crossing points of dispersive branches leading to saddle-point like features with quasi-constant energy along (0, 0.5, 0), (0.5, 0, 0) and equivalent lines in the Brillouin zone. While not quantitatively exact, the spin-wave calculation using DFT parameters gives with no adjusted parameters a quite good description of the measured results.

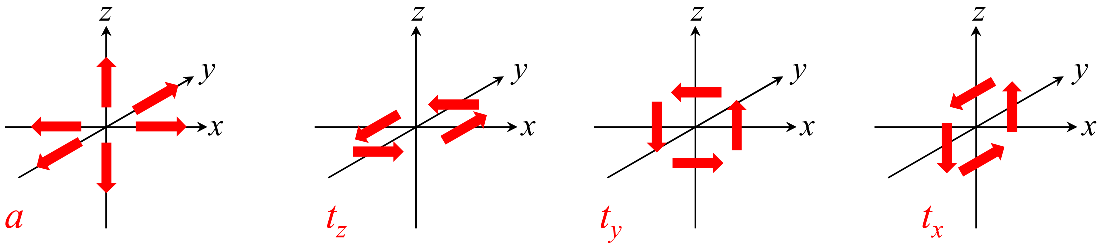

$$\begin{pmatrix}\alpha_{11}&0&0\\0&\alpha_{11}&0\\0&0&\alpha_{11}\end{pmatrix}\quad\begin{pmatrix}0&\alpha_{12}&0\\-\alpha_{12}&0&0\\0&0&0\end{pmatrix}\quad\begin{pmatrix}0&0&\alpha_{13}\\0&0&0\\-\alpha_{13}&0&0\end{pmatrix}\quad\begin{pmatrix}0&0&0\\0&0&\alpha_{23}\\0&-\alpha_{23}&0\end{pmatrix}$$

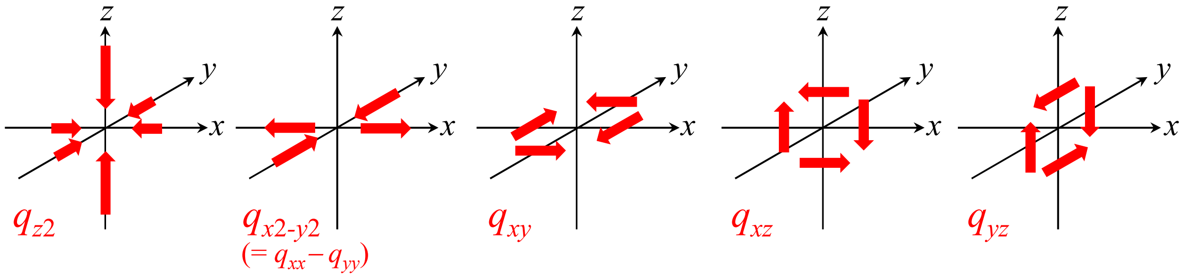

$$\begin{pmatrix}\alpha_{11}&0&0\\0&\alpha_{11}&0\\0&0&-2\alpha_{11}\end{pmatrix}\quad\begin{pmatrix}\alpha_{11}&0&0\\0&-\alpha_{11}&0\\0&0&0\end{pmatrix}\quad\begin{pmatrix}0&\alpha_{12}&0\\\alpha_{12}&0&0\\0&0&0\end{pmatrix}\quad\begin{pmatrix}0&0&\alpha_{13}\\0&0&0\\\alpha_{13}&0&0\end{pmatrix}\quad\begin{pmatrix}0&0&0\\0&0&\alpha_{23}\\0&\alpha_{23}&0\end{pmatrix}$$

Supplementary Figure 6: Nine types of magnetic multipole moments and corresponding magnetoelectric tensors. Here, a, $\mathbf{t}$, and q_{ij} represent magnetoelectric monopole, toroidal, and quadrupole moments, respectively. Solid red arrows represent magnetic dipole moments. The corresponding magnetoelectric tensors of each multipole are also indicated.

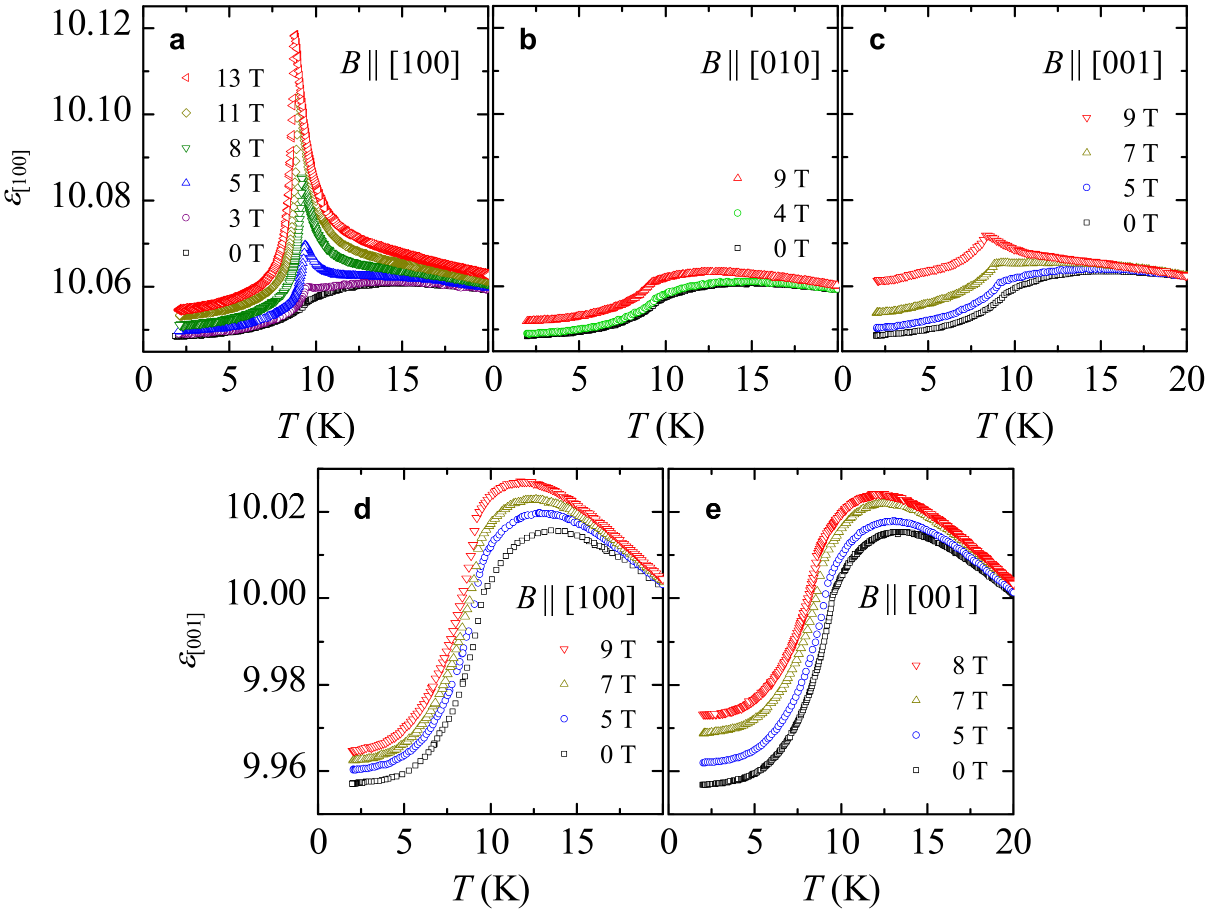

Supplementary Figure 7: Magnetodielectric properties in various measurement configurations. Temperature (T) profiles of the dielectric constants along the [100] direction ($\varepsilon_{[100]}$) in various magnetic fields (B) parallel to the [100] (**a**), [010] (**b**), and [001] (**c**) directions and those of $\varepsilon_{[001]}$ in B parallel to the [100] (**d**) and [001] (**e**) directions. For completeness, we plot in (**a**) the identical data used for Fig. 3 of the main text. The field-induced divergent peak toward T_N is observed only in $\varepsilon_{[100]}$ under B||[100].

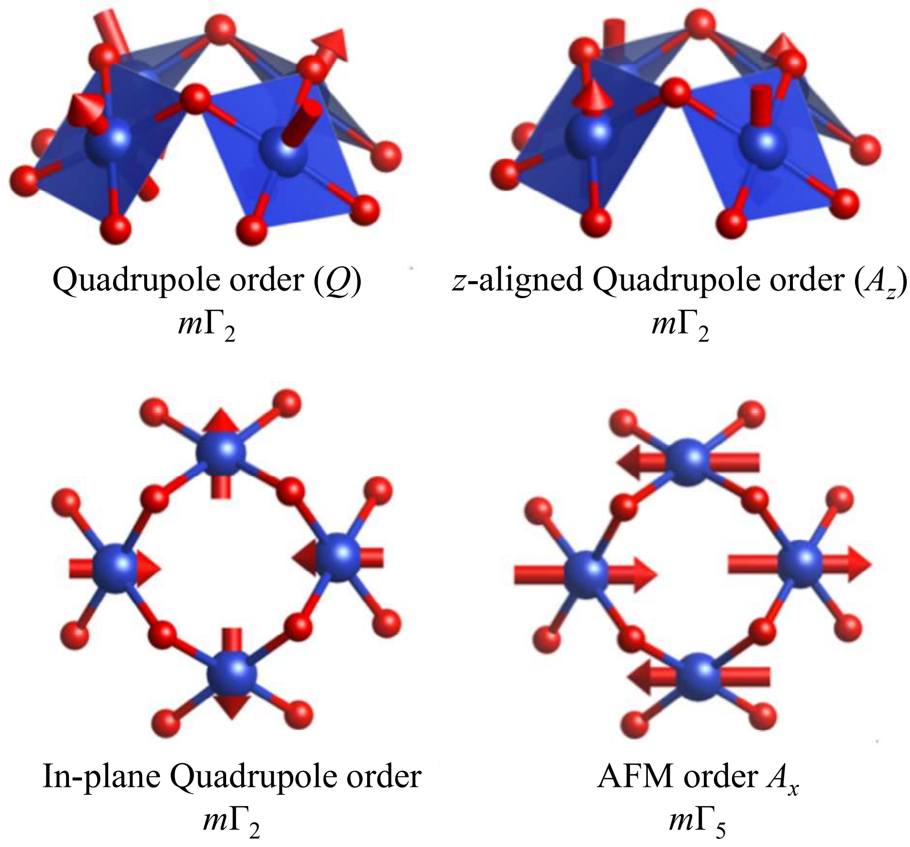

Supplementary Figure 8: Magnetic ordering and corresponding irreducible representation. Here, the blue and red balls represent Cu and O ions, respectively. The red allows denote the magnetic dipole moment.

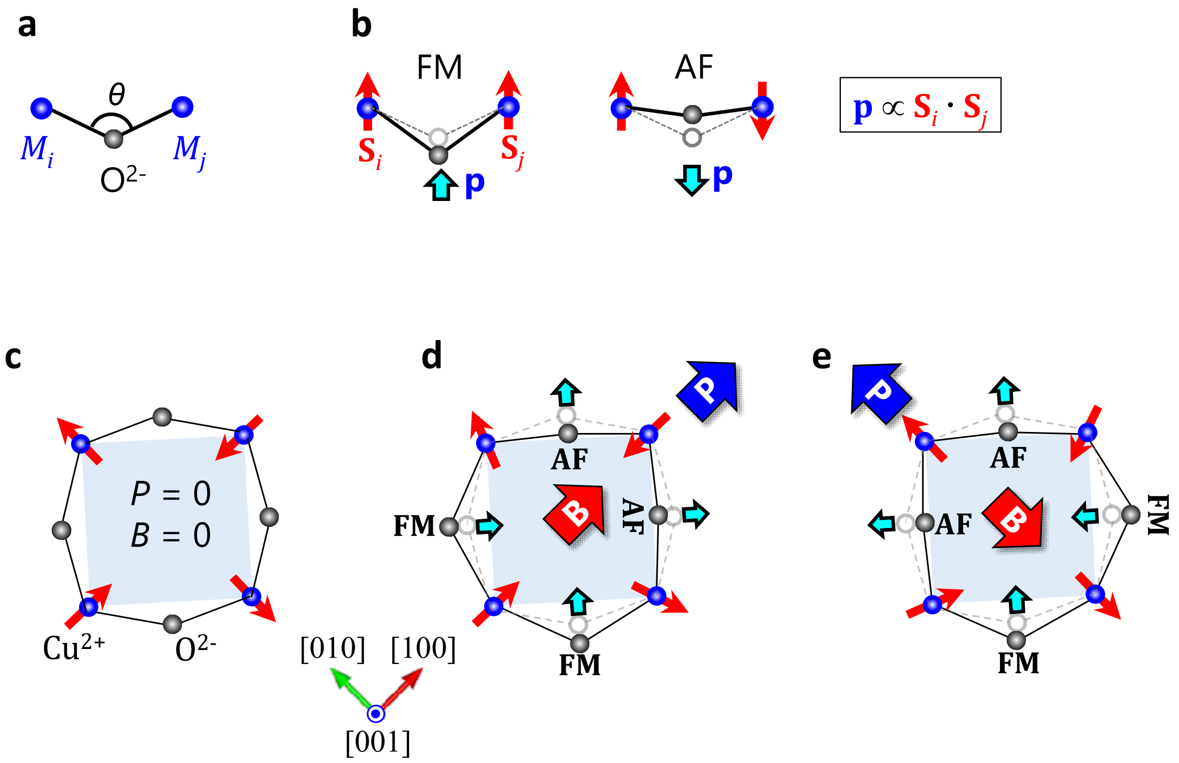

Supplementary Figure 9: Magnetostriction mechanism of magnetoelectric activity. a, Two magnetic cations M_i and M_j connected by a ligand with a bond angle θ. **b**, When S_i and S_j align ferromagnetically (FM) [antiferromagnetically (AF)] upon magnetic ordering, the ligand position will shift to downward (upward) to make the bond angle close to 90° (180°) which stabilizes the FM (AF) superexchange interactions. This magnetostriction mechanism leads to a local electric dipole p whose direction depends on the sign of product of $\mathbf{S}_i$ and $\mathbf{S}_j$. (i.e., $\mathbf{p} \propto \mathbf{S}_i \cdot \mathbf{S}_j$). **c**, The c-axis view of a Cu square-cupola cluster at B = 0, where in-plane spin components and O^{2-} atoms associated with the strongest superexchange interaction J_1 are denoted. The local electric dipole through the magnetostriction mechanism does not appear because of $\mathbf{S}_i \cdot \mathbf{S}_j = 0$. **d**, Upon the application of a magnetic field along the [100] direction, a finite $\mathbf{S}_i \cdot \mathbf{S}_j$ emerges on each spin pair, which generates a local electric dipole. Summing the induced dipoles over all bonds, one can find that the electric polarization (**P**) is induced parallel to the magnetic field on the square cupola. **e**, From the same discussion, the [010] magnetic field produces **P** antiparallel to the field direction.

Supplementary Tables

Supplementary Table 1: Nuclear structure refinement of $Ba(TiO)Cu_4(PO_4)_4$ at 1.5 K.

$Ba(TiO)Cu_4(PO_4)_4$ at 1.5 K
Space group: $P42_12$
Lattice parameters: $a = b = 9.60759(19)$ Å
$c = 7.11120(16)$ Å
Density: 4.226 gcm^{-3}

Atom	site	*x*	*y*	*z*
Ba	2a	0	0	0
Cu	8g	0.26710(22)	0.98794(29)	0.40399(31)
Ti	2c	0.5	0	0.98458(185)
P	8g	0.28174(37)	0.18954(37)	0.74268(69)
O(1)	8g	0.34889(37)	0.13491(37)	0.55742(49)
O(2)	8g	0.12406(32)	0.16086(33)	0.73507(59)
O(3)	8g	0.33503(31)	0.11131(37)	0.90913(51)
O(4)	2c	0.5	0	0.21687(95)
O(5)	8g	0.31727(33)	0.34579(33)	0.75860(70)

Atom	β_{11}	β_{22}	β_{33}	$\delta\beta_{11}$	$\delta\beta_{22}$	$\delta\beta_{33}$
Ba	24.8	24.8	-20.4	7.7	7.7	19.6
Cu	-4.4	28.6	121.2	3.5	4.0	8.4
Ti	32.7	32.7	244.0	12.6	12.6	42.8
P	-4.0	-4.0	122.2	3.3	3.3	11.7
O(1)	-4.7	28.0	74.5	6.5	7.5	9.7
O(2)	21.6	27.4	73.1	5.3	5.3	12.4
O(3)	19.1	20.8	27.1	6.0	6.2	10.0
O(4)	18.6	18.6	84.0	5.7	5.7	20.3
O(5)	21.2	8.5	210.8	5.5	5.1	18.1

The uncertainties in the refinement are given in brackets. The anisotropic thermal factors β are dimensionless with uncertainty in the fit parameter given by $\delta\beta$. The values of β and $\delta\beta$ above have been multiplied by 10^4. The β-parameters with negative values are not physical and within the uncertainty can be considered as very small instead.

Supplementary Table 2: Character table of the little group G_k for a commensurate magnetic propagation wave vector k = (0, 0, 0.5).

	1	2 0,0,z	2(0,½,0) ¼,*y*,0	2(½,0,0) *x*,¼,0	2 *x*,*x*,0	2 *x*,−*x*,0	4^- ½,0,*z*	4^+ 0, ½,z
Γ_1	1	1	1	1	1	1	1	1
Γ_2	1	1	1	1	−1	−1	−1	−1
Γ_3	1	1	−1	−1	1	1	−1	−1
Γ_4	1	1	−1	−1	−1	−1	1	1
Γ_5	$\begin{pmatrix}1 & 0\\0 & 1\end{pmatrix}$	$\begin{pmatrix}-1 & 0\\0 & -1\end{pmatrix}$	$\begin{pmatrix}1 & 0\\0 & -1\end{pmatrix}$	$\begin{pmatrix}-1 & 0\\0 & 1\end{pmatrix}$	$\begin{pmatrix}0 & 1\\1 & 0\end{pmatrix}$	$\begin{pmatrix}0 & -1\\-1 & 0\end{pmatrix}$	$\begin{pmatrix}0 & -1\\1 & 0\end{pmatrix}$	$\begin{pmatrix}0 & 1\\-1 & 0\end{pmatrix}$

Supplementary Table 3: Matrices of the generators of space group $P42_121'$ in the representations spanned by magnetic order parameters (F, A, *Q*) and electric polarization (P).

	1	2 0,0,*z*	4^+ 0,1/2,*z*	2(0,1/2,0) 1/4,*y*,0	1′	IR	SPG
F_x F_y	$\begin{pmatrix}1 & 0\\0 & 1\end{pmatrix}$	$\begin{pmatrix}-1 & 0\\0 & -1\end{pmatrix}$	$\begin{pmatrix}0 & -1\\1 & 0\end{pmatrix}$	$\begin{pmatrix}-1 & 0\\0 & 1\end{pmatrix}$	$\begin{pmatrix}-1 & 0\\0 & -1\end{pmatrix}$	$m\Gamma_5$ (A_1, A_4)	$P2_12_1'2'$
F_z	1	1	1	−1	−1	$m\Gamma_3$	$P42_1'2'$
A_x A_y	$\begin{pmatrix}1 & 0\\0 & 1\end{pmatrix}$	$\begin{pmatrix}-1 & 0\\0 & -1\end{pmatrix}$	$\begin{pmatrix}0 & -1\\1 & 0\end{pmatrix}$	$\begin{pmatrix}1 & 0\\0 & -1\end{pmatrix}$	$\begin{pmatrix}-1 & 0\\0 & -1\end{pmatrix}$	$m\Gamma_5$ (A_2, A_3)	$P2_12_1'2'$
A_z	1	1	−1	−1	−1	$m\Gamma_2$	$P4'2_12'$
Q	1	1	−1	−1	−1	$m\Gamma_2$	$P4'2_12'$
P_x P_y	$\begin{pmatrix}1 & 0\\0 & 1\end{pmatrix}$	$\begin{pmatrix}-1 & 0\\0 & -1\end{pmatrix}$	$\begin{pmatrix}0 & -1\\1 & 0\end{pmatrix}$	$\begin{pmatrix}-1 & 0\\0 & 1\end{pmatrix}$	$\begin{pmatrix}1 & 0\\0 & 1\end{pmatrix}$	Γ_5 Γ_5	$P42_12$
P_z	1	1	1	−1	1	Γ_3	$P42_12$

1′ denotes the time reversal operator. Labels of irreducible representation (IR) are taken from the ISODISTORT program[2]. The symmetrically lowered space group (SPG) according to Bilbao Crystallographic Server[3–5] is also listed.

Supplementary Note 1: Expression of Magnetoelectric Multipole and Corresponding Magnetoelectric Tensor

To discuss the magnetoelectric (ME) properties of the monopole, toroidal, and quadrupole moments, the explicit expression of these entities is very important. According to refs. 6 and 7, the magnetic interaction energy H_{int} of magnetization density $\boldsymbol{\mu}(\boldsymbol{r})$ with an inhomogeneous magnetic field $H(\boldsymbol{r})$ can be written as a multipole expansion in powers of field gradients calculated at certain arbitrary point $\boldsymbol{r} = 0$ up to the first order:

$$\begin{aligned} H_{\text{int}} &= -\int \boldsymbol{\mu}(\mathbf{r})\cdot\mathbf{H}(\mathbf{r})d^3\mathbf{r} \\ &= -\int \boldsymbol{\mu}(\mathbf{r})\cdot\mathbf{H}(0)d^3\mathbf{r} - \int r_i\mu_j(\mathbf{r})\partial_i H_j(0)d^3\mathbf{r} \\ &= -\mathbf{m}\cdot\mathbf{H}(0) - a(\nabla\cdot\mathbf{H})_{r=0} - \mathbf{t}\cdot[\nabla\times\mathbf{H}]_{r=0} - q_{ij}\left(\partial_i H_j + \partial_j H_i\right)_{r=0}. \end{aligned} \quad (1)$$

The first term describes the usual interaction between magnetic moment $\mathbf{m} = \int\boldsymbol{\mu}(\mathbf{r})d^3\mathbf{r}$ and a magnetic field $H(0)$. In the first order terms, there are three types of multipole moments: the monopole moment a, the toroidal moment $\mathbf{t}$, and the quadrupole moment q_{ij}, which are defined as:

$$a = \frac{1}{3}\int \mathbf{r}\cdot\boldsymbol{\mu}(\mathbf{r})d^3\mathbf{r}. \quad (2)$$

$$\mathbf{t} = \frac{1}{2}\int \mathbf{r}\times\boldsymbol{\mu}(\mathbf{r})d^3\mathbf{r}. \quad (3)$$

$$q_{ij} = \frac{1}{2}\int\left[r_i\mu_j(\mathbf{r}) + r_j\mu_i(\mathbf{r}) - \frac{2}{3}\delta_{ij}r\cdot\boldsymbol{\mu}(\mathbf{r})\right]d^3\mathbf{r}. \quad (4)$$

All the moments change the sign under time reversal and space inversion. This broken symmetry is a necessary condition for the appearance of the linear ME effect. Therefore, a system with these ME multipole moments potentially shows the linear ME effect. For a localized spin system, these equations can be written as:

$$a = \frac{g}{3}\mu_B\sum_n \mathbf{r}_n\cdot\mathbf{S}_n. \quad (5)$$

$$\mathbf{t} = \frac{g}{2}\mu_B\sum_n \mathbf{r}_n\times\mathbf{S}_n. \quad (6)$$

$$q_{ij} = \frac{g}{2}\mu_B\sum_n\left[r_{ni}S_{nj} + r_{nj}S_{ni} - \frac{2}{3}\delta_{ij}\mathbf{r}_n\cdot\mathbf{S}_n\right]. \quad (7)$$

Here, n represents a label of the individual spins $\mathbf{S}_n$ at the position $\mathbf{r}_n$.

In the free energy expansion, the linear ME effect can be described by the term $\alpha_{ij}E_iH_j$, where α_{ij} is the 3×3 matrix ME tensor. The tensor α_{ij} can be decomposed into a pseudoscalar, a vector, and a symmetric traceless tensor,

$$F_{ME} = -\tilde{a}(\mathbf{E}\cdot\mathbf{H}) - \tilde{\mathbf{t}}\cdot[\mathbf{E}\times\mathbf{H}] - \tilde{q}_{ij}\left(E_iH_j + E_jH_i\right). \quad (8)$$

From the comparison of this equation with Supplementary Eq. 1, one can find the relationship between the ME multipole moment and the associated ME tensor. In Supplementary Fig. 6, we schematically illustrate nine types of spin arrangements which have the abovementioned multipole moments and associated ME tensor. The spin arrangement of each Cu square cupola in $Ba(TiO)Cu_4(PO_4)_4$ described in the next section has only a quadrupole moment, which is composed of a large fraction of q_{x2-y2} and a small fraction of q_{xy}.

Supplementary Note 2: Description of Magnetic Quadrupole Order

We present a detailed description of the alignment of the magnetic quadrupoles on Cu square cupolas. As can be found from Supplementary Table 2, the magnetic symmetry of each square cupola in the proposed Γ_3 magnetic structure is 4′, because 4′ rotation axis is located at the center of each square cupola. In terms of symmetry, this allows two types of quadrupole moments, q_{x2-y2} and q_{xy} (see Supplementary Fig. 6), where x and y denote the local coordinate of the given square cupola, which are approximately parallel to the [100] and [010] axes of the global coordinate (see Supplementary Fig. 1). To avoid complexity, we neglect this slight canting (~2.9°) of the x (y) axis from the [100] ([010]) direction. The ordering manner of these quadrupole moments in a square cupola layer can be understood by applying the 2_1 symmetry operation that connects square cupola A and B. As can be seen in Supplementary Fig. 1, upon the application of this symmetry operation, q_{x2-y2} does not change the sign while q_{xy} does. This means that only the q_{x2-y2} quadrupole moment aligns uniformly within the *ab*-plane (i.e., uniform quadrupole order). This is consistent with the overall symmetry 4′22′ that allows for a pure q_{x2-y2} quadrupole moment. Given the neutron diffraction results that the individual square cupola has the almost pure q_{x2-y2} component and negligibly small q_{xy}, together with the fact that the leading exchange interaction is intra square cupola exchange interaction J_1, the macroscopic dielectric response which is experimentally observed can be mainly ascribed to a summation of the ME activity due to the q_{x2-y2} quadrupole moment on each square cupola.

Supplementary Note 3: Analysis of Neutron Diffraction Measurements

To determine the nuclear and magnetic structures of $Ba(TiO)Cu_4(PO_4)_4$ we have performed neutron diffraction measurements using the time-of-flight diffractometer WISH at ISIS on a 8.1 g powder sample[8]. Data was collected between 1.5 and 20 K with typical counting times of around 25 min per temperature. The detector system of WISH allows for collection of high-resolution data covering a wide range of *d*-spacing with varying resolution. In our analysis we fit the data for $0.7 < d < 18$ Å. This is highly advantageous as it allows for accurate structural analysis and at the same time covers large enough *d*-spacing (small enough

|**Q**|) to examine the magnetic structure. In our analysis we have simultaneously refined the data using Fullprof[9] over the entire range to a model including nuclear and magnetic structures. We find traces of impurities in our spectra; however, the composition of these could not be identified and are left unrefined.

We first consider the crystallographic structure of $Ba(TiO)Cu_4(PO_4)_4$ in the $P42_12$ space group. We find excellent agreement with previous report[10]. In Supplementary Table 1 we present our results of the structural refinement at 1.5 K. An example of the refinement is shown in Supplementary Fig. 2. The rather complicated structure consists of corrugated CuO_4 square planes separated by Ba, PO_4 tetrahedra and tetragonal TiO_{1+4} pyramids shown in Fig. 1 of the main article. The lattice parameters remain unchanged within the 1.5 to 20 K temperature range studied.

We next turn our attention to the magnetic structure refinement at 1.5 K, which has a commensurate magnetic propagation wave vector $\mathbf{k} = (0,0,0.5)$. Group representation theory can be used to classify the possible magnetic structures emanating from the paramagnetic group from which the magnetic order emerges. In the present case we use Basireps[9] to perform such a decomposition and outline these results here. The little group $G_\mathbf{k}$ contains the following symmetry operators of the paramagnetic space group ($P42_12$) given in Supplementary Table 2. The magnetic representation can be decomposed to a direct sum of irreducible representations (IR) as, $\Gamma_{mag}\ (8g) = 3\Gamma_1 + 3\Gamma_2 + 3\Gamma_3 + 3\Gamma_4 + 6\Gamma_5^{(2)}$. With the exception of $\Gamma_5^{(2)}$ which is two-dimensional, the other IRs are purely one-dimensional. The character table for $G_\mathbf{k}$ is given in Supplementary Table 2. The final Fourier coefficients obtained from the basis function calculated for the Γ_3 IR for the Cu sites resolved along crystallographic axes are,

$1.(u,v,w)$; $2.(\bar{u},\bar{v},w)$; $3.(v,\bar{u},\bar{w})$; $4.(u,\bar{v},w)$

$5.(\bar{v},u,\bar{w})$; $6.(\bar{u},v,w)$; $7.(\bar{v},\bar{u},\bar{w})$; $8.(v,u,\bar{w})$

The parameters u, v and w are free parameters of the possible magnetic structure which are to be determined experimentally. Equivalent calculations can be made for the other IRs in Γ_{mag}; however, by fitting the measured powder patterns we find that Γ_3 gives the best agreement. The case of $\Gamma_5^{(2)}$ can produce a magnetic structure with an amplitude modulated moment, which seems unlikely. Moreover, this IR leads to 12 basis functions which cannot be reliably refined using our data.

Within the Γ_3 representation, we find two solutions which give similar quality of fit to the observed magnetic pattern. We shall label these as $\Gamma_3(1)$ and $\Gamma_3(2)$ whose coefficients are $(u,v,w) = (0.49(1),0.36(2),0.58(2))$ and $(0.48(1),0.1(3),-0.64(2))$, respectively. The magnetic structures of the two models are illustrated in Supplementary Fig. 3. In the case of $\Gamma_3(1)$ the moments are confined approximately in the plane of the CuO_4. Conversely, in the $\Gamma_3(2)$ model, the moments are approximately perpendicular to the CuO_4 planes and form an

up-down-up-down type arrangement within a Cu_4O_{16} square cupola cluster. The $\Gamma_3(2)$ model corresponds to the magnetic structure described in the main article. The *v* component of the magnetic moment cannot be resolved accurately in our measurements, fixing this to zero does not alter the quality of the fit.

Supplementary Figure 4 shows a fit of the $\Gamma_3(1)$ and $\Gamma_3(2)$ models to the neutron powder diffraction patterns of $Ba(TiO)Cu_4(PO_4)_4$ obtained at 1.5 K. The Rietveld refinement of the magnetic structure is restricted to $d > 2.5$ Å to avoid excessive peak overlap at smaller *d*-spacings where owing to the magnetic form factor the magnetic peaks are also greatly reduced. The magnetic *R*-factor for refinement of all banks is found to be 18.5% for $\Gamma_3(1)$ and 11.5% for $\Gamma_3(2)$. Therefore, the $\Gamma_3(2)$ model is the most consistent with our measurements.

Supplementary Note 4: Landau Symmetry Analysis of Magnetoelectric Effects

In order to characterize the ME effect, we discuss the group theory analysis in the framework of Landau theory of phase transitions[11] as considering $P42_12$ space group, which is non-polar but non-centrosymmetric. Similar studies on the ME effect in $P\bar{4}2_1m$ space group can be found in refs. 12 and 13. For the simplicity, we take into account the single layer of $Ba(TiO)Cu_4(PO_4)_4$ structure and neglect weak inter-layer magnetic interactions. The parent $P42_121'$ magnetic space group has eight symmetry operations shown in the first row of Supplementary Table 2 plus time-reversal (1′), whereas a magnetic ordering lowers symmetry into a magnetic space group. Here, we define FM order parameter **F**, AFM order parameter **A**, and quadrupole order parameter *Q* (see Supplementary Fig. 8). The components of the magnetic order parameters **M** = **F**, **A**, *Q* and of the electric polarization **P** transform as following the transformation rules given in Supplementary Table 3. Note that the quadrupole order parameter *Q* belongs to the same irreducible representation as A_z, so that A_z will be represented by *Q* henceforth. (Experimental results have revealed that the spin direction in the quadrupole configuration is approximately perpendicular to the CuO_4 plane, but the direction is not important from the symmetrical point of view.)

By means of these transformation rules, we can express the thermodynamic free energies in terms of all the possible ME coupling terms of the form $P{\cdot}M^2$ which are invariant under symmetry operations:

$$F_{\mathrm{ME}} = c_1\left(P_xF_zF_y - P_yF_zF_x\right) + c_2\left(P_yQF_y - P_xQF_x\right) \qquad \text{(S9)}$$

Here, c_1 and c_2 are constants. To analyze the ME response, we take into account the usual dielectric energy, $F_{\mathrm{DE}} = -\mathbf{P}^2/\chi$, where χ is the dielectric susceptibility, hereafter set as 1. **P** is then evaluated at the minima of $F = F_{\mathrm{ME}} + F_{\mathrm{DE}}$, when $\partial F/\partial P_i = 0$ ($i = x, y, z$), ending up with

$$
\begin{aligned}
P_x &= c_1 F_z F_y - c_2 Q F_x, \\
P_y &= -c_1 F_z F_y + c_2 Q F_y, \\
P_z &= 0.
\end{aligned}
\tag{S10}
$$

Here, P_x and P_y components originate from magnetic cross coupling terms. Assuming the quadrupole magnetic ground state, the application of a magnetic field along the *x* and *y* direction will yield the ferromagnetic order and the polarization along the *x* and *y* direction, respectively. This is consistent with the ME tensor discussed in the main text.

Supplementary References

1. Toth, S., and Lake, B. Linear spin wave theory for single-Q incommensurate magnetic structures. *J. Phys.: Condens. Matter* **27**, 166002 (2015)
2. Campbell, B. J., Stokes, H. T., Tanner E. E. & Hatch D. M. ISODISPLACE: a web-based tool for exploring structural distortions. *J. Appl. Cryst.* **39**, 607-614 (2006).
3. Aroyo, M. I. *et al*. Crystallography online: Bilbao Crystallographic Server. *Bulg. Chem. Commun.* **43**, 183-197 (2011).
4. Aroyo, M. I. *et al*. Bilbao Crystallographic Server: I. Databases and crystallographic computing programs. *Z. Kristallogr.* **221**, 15-27 (2006).
5. Aroyo, M. I., Kirov, A., Capillas, C., Perez-Mato J. M. & Wondratschek, H. Bilbao Crystallographic Server. II. Representations of crystallographic point groups and space groups. *Acta Crystallogr. A* **62**, 115-128 (2006).
6. Spaldin, N. A., Fiebig, M. & Mostovoy, M. The toroidal moment in condensed-matter physics and its relation to the magnetoelectric effect. *J. Phys.: Condens. Matter* **20**, 434203 (2008).
7. Spaldin, N. A., Fechner, M., Bousquet, E., Balatsky, A. & Nordström, L. Monopole-based formalism for the diagonal magnetoelectric response. *Phys. Rev. B* **88**, 094429 (2013).
8. Chapon, L. C. *et al*. Wish: The new powder and single crystal magnetic diffractometer on the second target station. *Neutron News* **22**, 22–25 (2011).
9. Rodriguez-Carvajal, J. Recent advances in magnetic structure determination by neutron powder diffraction. *Physica B: Condensed Matter* **192**, 55-69 (1993).
10. Kimura, K., Sera, M. & Kimura, T. A^{2+} Cation Control of Chiral Domain Formation in $A(TiO)Cu_4(PO_4)_4$ (*A* = Ba, Sr). *Inorg. Chem.* **55**, 1002–1004 (2016).
11. Landau, L. D. & Lifshitz, E. M. *Statistical Physics*, Part I (Pergamon, Oxford, 1980).
12. Yamauchi, K., Barone, P. & Picozzi, S. Theoretical investigation of magnetoelectric effects in $Ba_2CoGe_2O_7$, *Phys. Rev. B* **84**, 165137 (2011).
13. Yamauchi, K., Oguchi, T. & Picozzi, S. Ab-initio prediction of magnetoelectricity in infinite-Layer $CaFeO_2$ and $MgFeO_2$. *J. Phys. Soc. Jpn.* **83**, 094712 (2014).
